# Prevention and Control of Fish-borne Zoonotic Trematodes in Fish Nurseries, Vietnam

**DOI:** 10.3201/eid1809.111076

**Published:** 2012-09

**Authors:** Jesper Hedegaard Clausen, Henry Madsen, K. Darwin Murrell, Phan Thi Van, Ha Nguyen Thi Thu, Dung Trung Do, Lan Anh Nguyen Thi, Hung Nguyen Manh, Anders Dalsgaard

**Affiliations:** University of Copenhagen, Copenhagen, Denmark (J.H. Clausen, H. Madsen, K.D. Murrell, A. Dalsgaard);; Research Institute for Aquaculture No.1, Bac Ninh, Vietnam (P.T. Van, H.N.T. Thu);; National Institute of Malariology, Parasitology, and Entomology, Hanoi, Vietnam (D.T. Do);; National Institute of Veterinary Research, Hanoi (L.A.N. Thi);; and Vietnam Academy of Science and Technology, Hanoi (H.N. Manh)

**Keywords:** fishborne zoonotic trematodes, aquaculture, prevention and control, Opisthorchiidae, Heterophyidae, parasites, zoonoses, Vietnam

## Abstract

Worldwide, >18 million persons were infected with fish-borne zoonotic trematodes in 2002. To evaluate the effectiveness of interventions for reducing prevalence and intensity of fish-borne zoonotic trematode infections in juvenile fish, we compared transmission rates at nurseries in the Red River Delta, northern Vietnam. Rates were significantly lower for nurseries that reduced snail populations and trematode egg contamination in ponds than for nurseries that did not. These interventions can be used in the development of programs for sustained control of zoonotic trematodes in farmed fish.

## Abstract 2

Reducing snails and trematode eggs in nursery ponds lowered trematode transmission among fish.

Liver and intestinal trematodes are major fish-borne zoonotic parasites of humans. The World Health Organization and the Food and Agriculture Organization of the United Nations estimate that >18 million persons were infected with fish-borne zoonotic trematodes (FZTs) in 2002 (*1**,**2*), and the World Health Organization recently added FZT infections to its list of emerging infectious diseases. FZTs are especially problematic in Asian countries, where fish are a main source of protein (*3*). They are highly prevalent in Vietnam, in cultured and wild-caught fish (*4**–**7*), and cause major concern for food safety (*8**,**9*).

In aquaculture systems, the main risk factors for FZT infection and transmission include contamination of pond environments with FZT eggs from infected hosts, i.e., humans, cats, dogs, pigs, and fish-eating birds. Factors that promote the diversity and population growth of snail intermediate hosts (families Thiaridae and Bithynidae) also increase risk (*10**–**14*). In Vietnam, a large proportion of aquaculture production, mainly of cyprinid fishes, originates from small family farms that integrate other agricultural activities. These integrated fish–livestock systems are called VAC systems (in Vietnamese, the word *vuon* means garden, *ao* means pond, and *chuong* means pigpen). Prevalence studies have demonstrated that in Vietnam, these integrated systems pose the highest risk for FZT transmission (*12**,**15*). The ponds become contaminated through use of animal, and occasionally human, fecal waste as pond fertilizer and through runoff water from pond banks and adjoining fields. Fish fry (newly hatched fish) are introduced into these ponds at about 2–3 days of age, and when they become juveniles (after 6 weeks), they are transferred to grow-out ponds. Efforts to control snail invasion and their population growth in fish ponds are usually minimal. FZT egg contamination of pond environments and FZT transmission are increased by the practice of feeding fish waste to domestic animals and by the cultural preference for eating raw or inadequately prepared fish dishes (*16*).

Recent findings from an investigation in northern Vietnam (*4**,**15**,**17*) revealed high rates of FZT transmission, especially in fish nurseries, where FZT prevalence among stocked FZT-free fish fry increased to 14.1%, 48.6%, and 57.8% after 1 week, 4 weeks, and when overwintered in ponds, respectively. The juvenile fish raised in nurseries are eventually transferred to grow-out farms, thereby potentially seeding a large number of grow-out farms with FZTs.

Our study objective was to evaluate the effectiveness of interventions to prevent FZT transmission in nurseries in northern Vietnam. Intervention goals were to reduce FZT egg contamination and to control snail populations in nursery ponds. The study is the first stage in developing a practical and sustainable program for prevention of FZT infections in aquaculture.

The project objective, planned interventions, and sampling procedures were approved by all participating institutions and their research committees in Vietnam. Risks, rights, and benefits were explained to all participating intervention farmers at the beginning of the project. Farmers were informed that they were free to withdraw from the study at any time. Persons from households associated with nonintervention nurseries were examined at the end of the study, and treatment was provided for free to those with egg counts indicating infection with trematodes. Thus, all household members received the same health benefits.

## Materials and Methods

### Study Area Design

**Figure 1:**
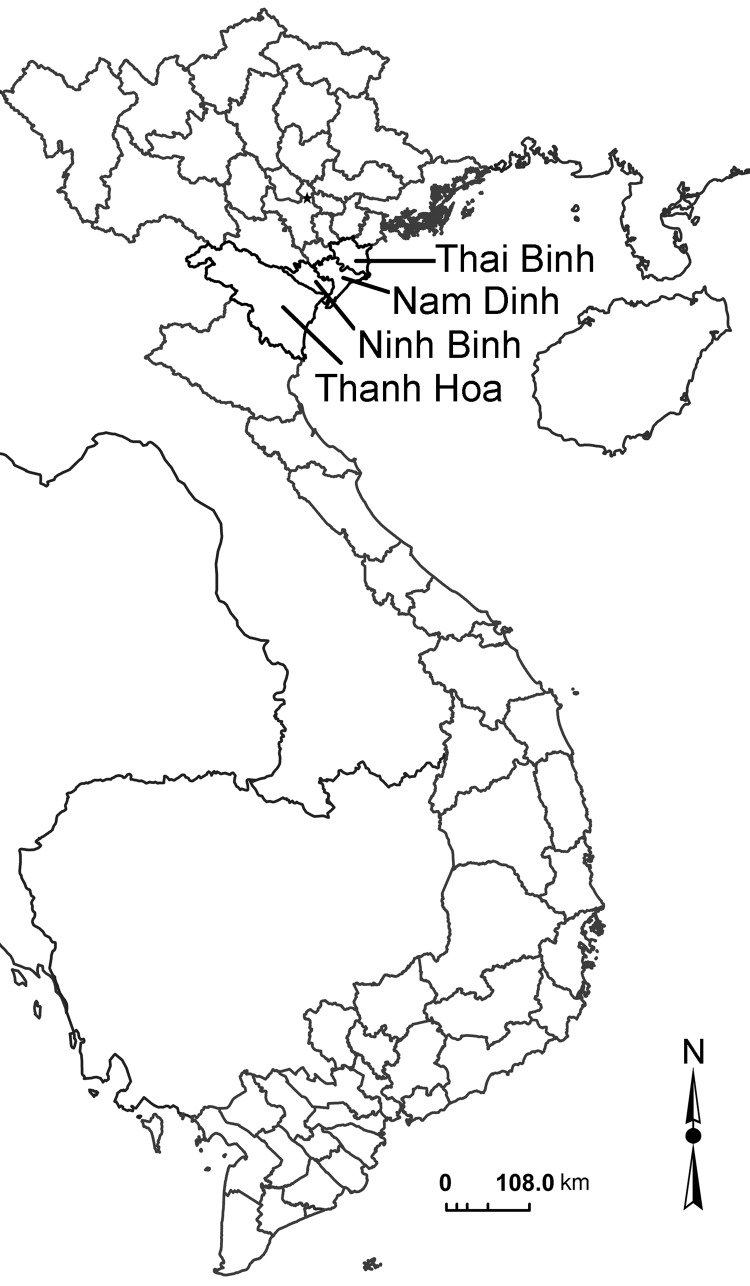
The 4 River Delta provinces of Vietnam (light gray borders).

The intervention study was conducted in 4 provinces in the Red River Delta of northern Vietnam (Thai Binh, Nam Dinh, Ninh Binh, and Thanh Hoa Provinces) (Figure 1). These provinces were selected because of their known high prevalence of FZT infections among humans, animals, and cultured fish (*8**,**9*). The study design was a parallel group design of nurseries: 1 intervention group and 1 nonintervention group. Nurseries were randomly selected from a list provided by the Department of Agriculture and Rural Development of each province. The selection criteria were as follows: small farms (<1,500 m^2^ of ponds), farms managed by household members, and nursery production of rohu (*Labeo rohita*) in an integrated fish–livestock system. Only 1 pond (study unit) per farm was selected for each intervention and nonintervention nursery. The 2 groups of nurseries were matched as closely as possible by type of production system, location, and size. The fry supplied from hatcheries to the nurseries were confirmed free of FZT metacercariae. Rohu, the fish species produced in all selected nurseries is a well-documented host for FZTs (*15*).

In 2009, a total of 18 intervention nurseries and 18 nonintervention nurseries from the 4 provinces were included in the study. The intervention trial period was May–September 2009 and involved 1 production cycle of juvenile fish. Studies have shown this to be a period of high FZT transmission (*15*). In 2010, different groups of 6 intervention and 6 nonintervention nursery ponds from Nam Dinh and Ninh Binh Provinces were added to the trial (for a total of 24 in each group), which was conducted during May–September 2010.

### Interventions

**Figure 2:**
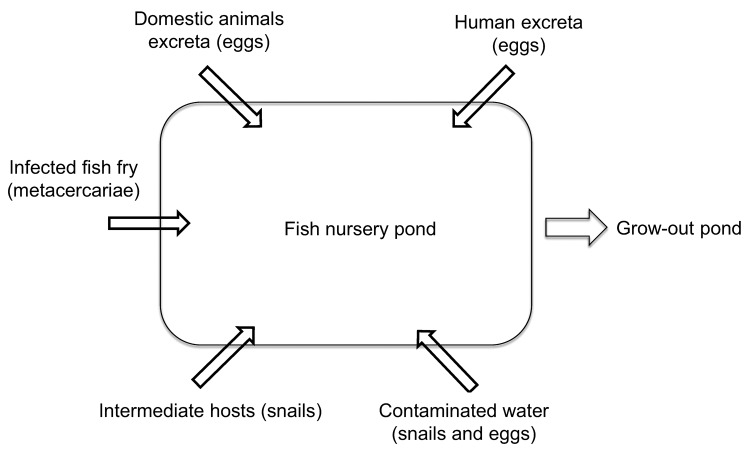
Main risk factors for transmission of fish-borne zoonotic trematodes in fish nurseries, Vietnam. Each risk factor (arrow pointing into pond) is also an intervention point.

The interventions were based on previously identified risk factors for FZT transmission in aquaculture (Figure 2) (*13**,**17*). In 2009 and 2010, we evaluated the combined effects of the interventions aimed at reducing environmental contamination with FZT eggs and reducing snail populations in ponds. All farmers for the nurseries selected for intervention participated in a training course on FZT biology and epidemiology. The intervention study objectives and design were explained, and the farmers were given specific instructions on the planned interventions for their individual nursery, determined on the basis of evaluations of risk factors associated with their nurseries. All farmers in the intervention group provided detailed information about the management practices they used to suppress snail populations and their habitat when preparing the nursery ponds before stocking and about the continuing management of the pond after stocking of fry.

At the start of the trial, the ponds at the intervention nurseries were emptied of water and dried for 5 days, during which time the top 3–5 cm of mud from the pond floor was removed to reduce the number of snails. Farmers were also instructed to remove all vegetation from the pond to reduce snail habitat. The banks of the ponds were lined with concrete to help control vegetation. Furthermore, 2 days before the ponds were refilled with water, aquatic vegetation within 3 m of each side of the water intake outside the pond was removed. A filter sock with a 5-mm mesh was fitted around the water inlet pipe to help reduce snail immigration into the pond.

To assess the effects of snail-control interventions, snail density was estimated before fish were stocked and during the nursery cycle. Samples were obtained in a scoop net, 0.25 m wide, cast 2 m off shore, and dragged perpendicularly back to the pond bank. Thus, the area sampled was 0.25 m × 2 m. A total of 5 such samples per pond were obtained. The samples were washed, and all live snails were brought to a field laboratory, where they were examined for cercarial infection according to procedures described by Dung et al. (*14*). Snail sampling was conducted before the intervention cycle and at 3, 6, and 9 weeks after stocking of fry during May–September of 2009 and 2010. Snails were identified by using taxonomic keys (*18**,**19*). Only snails that were potential hosts of FZTs were included in the analysis, i.e., species of the families Thiaridae (*Melanoides tuberculata*, *Sermyla tornatella*, *Thiara scabra*, and *Tarebia granifera*) and Bithynidae (mainly *Bithynia fuchsiana* and *Parafossarulus striatulus*).

Before the trials, all residents, workers, and immediate neighbors of the intervention nurseries were examined for trematode infections by medically trained staff. The Kato–Katz fecal egg count method was used (*20*). Two Kato–Katz smears of 41.7-mg fecal samples were independently examined under a microscope at 400× magnification. FZT eggs were counted and recorded as eggs per gram. All persons in whom trematode eggs were found were treated with praziquantel at 25 mg/kg, administered 3×/d for 1 day (total 75 mg/kg). During the 2009 and 2010 intervention trials, fecal examinations were repeated after 30 and 60 days. Residents and workers of nonintervention nurseries were examined for FZT infection at the end of the trials and offered free treatment with praziquantel if found to be infected with FZTs.

Domestic animals (dogs, cats, and pigs) from the households of the intervention nursery workers and the neighboring households were examined for FZT eggs according to the fecal examination procedure described by Lan Anh et al. (*21*). Animals in which eggs were found were given 1 dose of praziquantel at 40 mg/kg (*22**,**23*). Fecal examinations were repeated every 30 days during the production cycle, and animals with positive results were again treated.

Prevention of FZT egg contamination of the nursery ponds consisted of constructing 10–15 cm elevated cement barrier walls along the pond banks to keep surface water runoff from entering the pond. A 50-cm-high fine mesh net was placed around the ponds to keep dogs and cats out. In all intervention nurseries, deliberate discharge of liquid wastes from pigpens and human latrines into the pond as fertilizer was terminated by blocking drains with cement and bricks. During training sessions, farmers were instructed to deposit pig manure and human excreta fertilizer away from the pond to prevent fecal contamination from surface runoff water.

In addition to providing treatment for FZT- infected humans and animals, we also cautioned farmers and members of intervention households to not eat raw or inadequately cooked fish and to not feed uncooked fish to dogs, cats, or pigs. For intervention nurseries, a project team had regular contact at least weekly with the farmers during pond preparation and during the nursery production cycle. During these visits, they discussed experiences and answered questions about the interventions.

Farmers of nonintervention nurseries were informed that a survey was being conducted on nursery management practices and that examination of their juvenile fish was needed for the survey. These farmers were not asked to alter any of their routine practices during the study periods. At the end of the project, all household members from nonintervention nurseries were informed about FZTs and the interventions to reduce FZT transmission.

### Fish Sampling and Examinations

Before stocking of fry, 100 fry from the supplying hatchery were analyzed for metacercariae by the compression method (*24*). At 3, 6, and 9 weeks after stocking of the fry, 100 juvenile fish from each pond were randomly collected in a seine net. The fish were placed on ice and transported within 36 h to the laboratory, where they were maintained at 4°C for a maximum of 5 days until processed. Each whole fish was then ground with a small handheld homogenizer and transferred to a beaker for digestion in a pepsin solution (8 mL concentrated HCl and 6 g pepsin in 1,000 mL water) to release metacercariae (*17*). After digestion, the metacercariae were isolated by sediment washing and identified and counted by examination with a microscope. For each juvenile fish, the number of metacercariae was determined and expressed as intensity (total metacercariae/fish). Up to 10 randomly selected metacercariae from each infected fish were identified to the species level, and the rest of the metacercariae were sorted into >1 type groups: liver flukes (Opisthorchiidae), intestinal flukes (Heterophyidae), and nonzoonotic or dead metacercariae. Metacercariae were identified by using morphologic criteria (*11**,**17*). All metacercaria species recovered were pooled for statistical analyses.

### Data Analyses

Data were double-entered into an Access database (Microsoft Corporation, Redmond, WA, USA) and analyzed by using statistical analysis system software (STATA 11; StataCorp LP, College Station, TX, USA). In 2009, we excluded 1 intervention and 2 nonintervention nursery ponds from the analysis because of high numbers of juvenile fish. In 2010, we excluded another 2 intervention nurseries because the farmers failed to follow intervention protocols.

The number of juvenile fish examined for metacercariae and the area (m^2^) searched for snails were used as offsets for the statistical analysis. The analysis used was negative binomial regression (*25*) in a generalized linear model (log link function) among sampling times; interventions and the interaction between sampling times and interventions were entered as factors adjusting for clustering within ponds. The ancillary parameter (α) of the variance function (var = μ + α μ^2^) was estimated in a full maximum-likelihood estimation (*25*). The total number of infected juvenile fish was analyzed by using logistic regression with the same predictor as for metacercaria counts and adjusting for clustering within ponds. Furthermore, the numbers of host snails, infected host snails, and relative egg contamination potential were tested as predictors for infections in fish and snails. The relative egg contamination potential for the animals studied was estimated; i.e., actual fecal egg counts in humans, dogs, cats, and pigs were multiplied by the estimated daily feces production, which was 160, 99, 20, and 1,516 g, respectively (*21**,**26*). The egg numbers were summed for all humans and animals within a given household.

## Results

### Metacercariae in Fish

A total of 9,266 juvenile fish in 2009 and 5,911 in 2010 from intervention and nonintervention nurseries were examined for FZT metacercariae. The following FZT species were identified: *Clonorchis sinensis* (Opisthorchiidae), *Centrocestus formosanus*, *Haplorchis pumilio* (Heterophyidae), and *Haplorchis yokogawi* (Heterophyidae). Among the recovered metacercariae, 94.00% belonged to the family Heterophyidae, 0.05% were *C. sinensis*, and 5.95% could not be identified.

**Figure 3:**
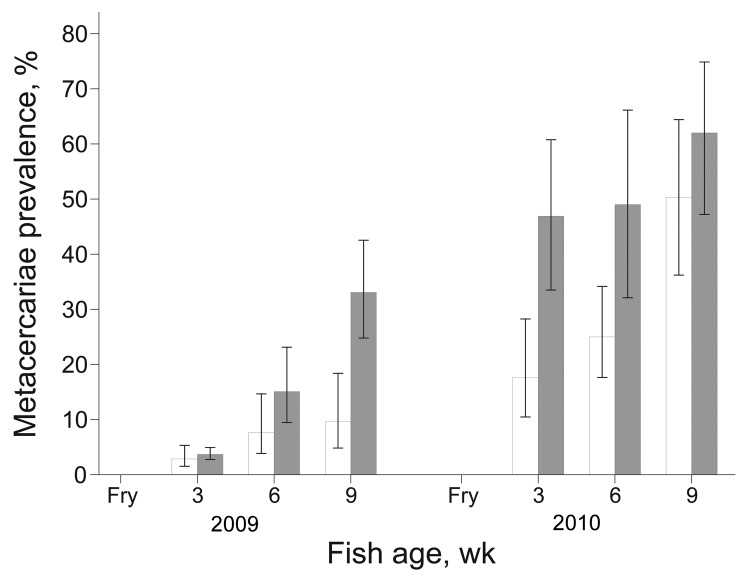
Mean prevalence of fish-borne zoonotic trematode metacercariae in juvenile fish from intervention (white bars) and nonintervention (gray bars) nurseries. Error bars indicate SEM.

**Figure 4:**
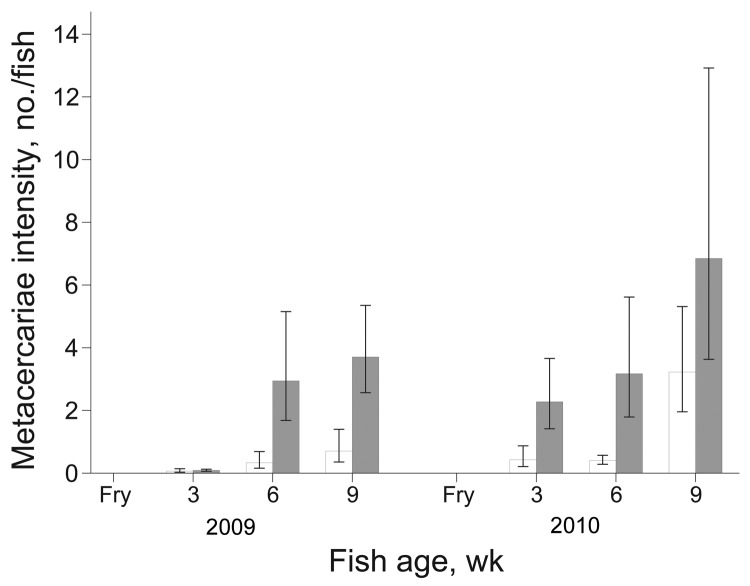
Mean intensity of fish-borne zoonotic trematode metacercariae/fish for juvenile fish from intervention (white bars) and nonintervention (gray bars) nurseries. Error bars indicate SEM.

During 2009, prevalence of FZT infection increased over time among juvenile fish from intervention and nonintervention ponds (Figure 3); however, the increase was significantly less for intervention farms (p<0.001). The difference in prevalence between intervention and nonintervention nursery ponds increased over time as shown by the significant interaction term (p<0.01) between intervention and time of sampling (Figure 3). At 3 weeks after stocking, the odds of infection in the intervention group were 0.77 (95% CI 0.19–3.12) of that in the nonintervention group; after 6 weeks, odds were 0.47 (95% CI 0.08–2.72); after 9 weeks, odds were only 0.22 (95% CI 0.04–1.14). The intensity of metacercariae infection increased over time (p<0.001) and was significantly lower among juvenile fish from the intervention nurseries (p<0.05) (Figure 4). The difference in intensity of metacercariae between intervention and nonintervention nurseries increased over time, and the interaction between the 2 types of nursery was significant (p<0.01). Thus, at 3 weeks after stocking, the intensity of infection in the intervention group was 0.75 (95% CI 0.14–3.82; p>0.05) of that in the nonintervention group; and after 6 and 9 weeks, the intensity was 0.11 (95% CI 0.02–0.68; p<0.05) and 0.19 (0.04–0.87; p<0.05), respectively.

In 2010, a similar pattern developed during the production cycle. At 3 weeks after stocking, the odds of infection in the intervention group were 0.24 (95% CI 0.05–1.23) of that in the nonintervention group, and after 6 and 9 weeks these odds were 0.35 (95% CI 0.07–1.79) and 0.62 (95% CI 0.12–3.19), respectively. Prevalence of infection increased considerably between 6 weeks and 9 weeks at intervention and nonintervention nurseries (Figure 3), consequently, the interaction between intervention and time of sampling was not significant.

In 2010, intensity of infection also increased over time (p<0.001) and the interaction term between intervention and time was significant (p<0.01). Thus, at 3 weeks after stocking, intensity of infection in the intervention group was 0.19 (95% CI 0.03–1.01; p>0.05) of that in the nonintervention group, and at 6 weeks it was 0.13 (95% CI 0.03–0.47; p<0.05). However, at 9 weeks, the intensity in juveniles from intervention nurseries increased to 0.47 of that in the nonintervention nurseries (95% CI 0.10–2.29; p<0.05) (Figure 4).

### Effect of Interventions on Snail Populations

**Figure 5:**
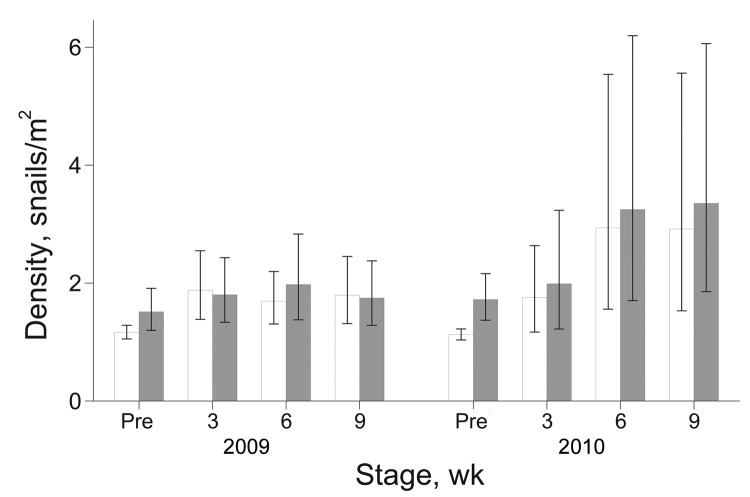
Geometric mean density of potential intermediate snail hosts per square meter in intervention (white bars) and nonintervention (gray bars) fish nursery ponds. Error bars indicate SEM. Pre, preintervention sampling of snails before stocking of fry.

During 2009–2010, a total of 30,590 snails were collected. The potential intermediate host of FZTs, i.e., snails of the families Thiaridae and Bithynidae, accounted for 57% (n = 17,478) and 0.04% (n = 133), respectively, of total collected snails. During these years, density of potential host snails (snails/m^2^) did not differ between intervention and nonintervention nurseries (Figure 5). The density of host snails was higher in the intervention group than in the nonintervention group when we adjusted for preintervention counts; differences were not significant when this adjustment was not made. However, for 2009, the odds of finding infection in host snails in the intervention group were 0.14 (95% CI 0.07–0.27; p<0.001) of that in the nonintervention group, and the interaction between time and treatment was not significant. For 2010, a significant interaction between time and treatment was found (p<0.05), but odds ratios and 95% CIs between intervention and nonintervention nurseries were <0.01 at all 3 sampling times (p<0.001).

### Excretion of Eggs by Humans and Domestic Animals

In 2009, providing treatment for humans in the farm households before and during the interventions reduced the prevalence of FZT eggs from 14.8% to 3.2% (Table); prevalence among comparable members of nonintervention households, who were tested at the end of the production cycle, was 14.2%. In 2010, FZT infections in humans were low or absent among members of intervention households and 12.5% among members of nonintervention households (Table).

**Table:** Prevalence and intensity of FZT infections, Vietnam*

Year and animal tested	Egg prevalence in nurseries†				No. eggs/g				Maximum no. eggs/g				Mean potential egg contamination index‡		
	Intervention§				Intervention§				Intervention§				Intervention§		
	None	Before	End		None	Before	End		None	Before	End		None	Before	End
2009															
Man	71 (14.1)	169 (14.8)	188 (3.2)		233.4	55.8	15.9		7,026.4	2,434.1	2,290.2		165,707	40,799	2,174
Cat	6 (50.0)	14 (50.0)	13 (15.4)		3.5	34.6	0.2		9.4	461.5	2.0		70	786	3
Dog	13 (30.8)	48 (18.8)	45 (15.6)		2.3	0.1	1		25.3	2.4	16.3		213	34	252
Pig	85 (1.2)	259 (0.8)	290 (0.3)		0	0	0		0.8	1.8	1.2		101	0	130
2010¶															
Man	32 (12.5)	70 (1.4)	66 (0)		24.7	0.2	0		431.7	12.0	0		25,324	0	0
Cat	0	7 (57.1)	2 (0)		0	12.9	0		0	60.0	0		0	80	0
Dog	2 (0)	17 (11.8)	15 (6.7)		0	3.8	0.1		0	49.0	1.0		0	1.073	0

*FZT, fish-borne zoonotic trematode; man, human (male or female); before, before intervention; end, end of production cycle. †No. hosts examined (% egg prevalence) ‡Number of eggs excreted by total numbers of final hosts (humans, cats, dogs, and pigs) to pond or pond surroundings. §FZT egg examinations at nonintervention farms were performed only at the end of the production cycle. The FZT infection status at nonintervention nurseries at the end of the production cycle is comparable with the infection status at intervention nurseries before the intervention was implemented. ¶Because of low FZT prevalence among pigs, pig data were not included in 2010 analyses.

In 2009, FZT prevalence and density among pigs from intervention and nonintervention nurseries were low (0.8% and <0.5 eggs/g, respectively), and no significant differences were found between intervention nurseries at the start and nonintervention nurseries at the end of the production cycle (Table). Because of the low prevalence among pigs, these data were not included in the 2010 analyses. During 2009, prevalence of FZT eggs among cats decreased from 50.0% to 15.4% and among dogs from 18.8% to 15.6%. In 2010, prevalence among cats decreased from 57.1% to 0 and among dogs, from 11.8% to 6.7% (Table).

Calculations of mean relative contamination index (no. eggs/household/day) for humans, cats, dogs, and pigs were based on total volumes of excreted feces and the actual egg intensities estimated (Table). Total potential egg contamination before the intervention did not differ significantly between intervention and nonintervention households. Total egg contamination potential did not correlate with prevalence and intensity of metacercaria infection in juvenile fish or with prevalence of infection in host snails.

## Discussion

Although prevalence and intensity of FZT infection in juvenile fish increased over time in intervention and nonintervention nurseries, the transmission rate was significantly reduced at intervention nurseries. Because of the need to ensure preharvest food safety in aquaculture products (*27*), the results of introducing risk-reduction measures into fish nurseries are encouraging. For the few previous attempts to prevent FZT infections in aquaculture in Thailand and Vietnam (*28**,**29*), success was limited.

The effect of interventions was similar in 2009 and 2010, except for week 9 in the 2010 cycle, when prevalence and intensity of infection in fish increased dramatically at intervention and nonintervention nurseries. We believe that flooding of the nurseries, which occurred in Nam Dinh in August 2010 after an unusually high tide, caused mixing of water from surrounding areas and contamination of the ponds with potentially infected snails. Sizes of snail populations show large variations among farms. In addition to local ecologic factors, this variation could also be influenced by flood-induced increases in snail distributions and migrations, leading to sudden rises in snail numbers. Because the increase in snail numbers occurred in intervention and nonintervention ponds, the increase was not associated with our control measures. Higher dikes around the ponds could have prevented the flooding of ponds, but the farmers cannot afford to build higher dykes. Flood surface water could also have increased contamination of ponds with FZT eggs from surrounding households and farms. Control of snails is the most challenging part of the interventions; the number of collected snails did not differ significantly between intervention and nonintervention nurseries. Future interventions should focus more on snail control, including new methods like biological and chemical control.

The treatment of FZT infections in humans, cats, dogs, and pigs clearly reduced fecal egg prevalence and density. Although humans had the highest potential for egg contamination, this potential might not reflect their role in sustaining FZT infections on the farm. Because cats and dogs shed most of the eggs into the environment, future interventions for reducing fecal egg contamination of the pond environment should focus on these hosts. In the nursery communities involved in this project, dog meat was often consumed; and at the time of slaughter, intestines are typically washed in the ponds. This practice could be a major source of egg contamination.

In addition to treatment to rid these hosts of infection, the prevention of latrine drainage into the ponds and more emphasis on training farmers about risk factors related to egg contamination should be beneficial. Training should emphasize the risks associated with free-ranging dogs and cats and their indiscriminate defecation near the ponds. The lack of effect on density of potential intermediate host snails between intervention and nonintervention nurseries suggests that the lower prevalence and intensity of FZTs in intervention nursery snails resulted from treatment of FZT infections in people and animals.

An integrated approach for controlling FZTs in aquaculture would include measures to prevent and control fecal pollution of ponds from animals and humans and reduction of infected snail hosts through better aquaculture management practices; however, the element of practicality cannot be ignored. Although pond management, snail control, and reduction of fecal pollution from latrine drainage can be incorporated reasonably well in a good management program, the treatment of FZT infections in humans and animals is more costly and difficult for the farm owner, family, and employees. It should be noted that the full effect of interventions will probably increase over repeated production cycles as a result of improved management skills of farmers and lower snail and egg concentrations in ponds and surrounding environments. Future efforts to develop a sustainable prevention and control strategy for FZTs should compare the effectiveness of interventions involving only pond management and snail control with and without treatment of FZT infection of household humans and animals.
